# Cucurbitacin B and Its Derivatives: A Review of Progress in Biological Activities

**DOI:** 10.3390/molecules29174193

**Published:** 2024-09-04

**Authors:** Wenzhe Nie, Yalan Wang, Xinlu Tian, Jinying Liu, Zhanhui Jin, Junjie Xu, Miaohai He, Qingkun Shen, Hongyan Guo, Tian Luan

**Affiliations:** 1 Key Laboratory of Natural Medicines of the Changbai Mountain, Ministry of Education, College of Pharmacy, Yanbian University, Yanji 133002, China; 2022051093@ybu.edu.cn (W.N.); 15981394661@163.com (Y.W.); 2022001112@ybu.edu.cn (J.L.); 2023010882@ybu.edu.cn (Z.J.); 2022010873@ybu.edu.cn (J.X.); 2022010885@ybu.edu.cn (M.H.); qkshen@ybu.edu.cn (Q.S.); 2 Department of Pharmacy, Shenyang Medical College, Shenyang 110034, China; 19915006959@163.com

**Keywords:** cucurbitacin B, structural modification, pharmacological action, dosage form improvement

## Abstract

The emergence of natural products has provided extremely valuable references for the treatment of various diseases. Cucurbitacin B, a tetracyclic triterpenoid compound isolated from cucurbitaceae and other plants, is the most abundant member of the cucurbitin family and exhibits a wide range of biological activities, including anti-inflammatory, anti-cancer, and even agricultural applications. Due to its high toxicity and narrow therapeutic window, structural modification and dosage form development are necessary to address these issues with cucurbitacin B. This paper reviews recent research progress in the pharmacological action, structural modification, and application of cucurbitacin B. This review aims to enhance understanding of advancements in this field and provide constructive suggestions for further research on cucurbitacin B.

## 1. Introduction

**Figure 1:**
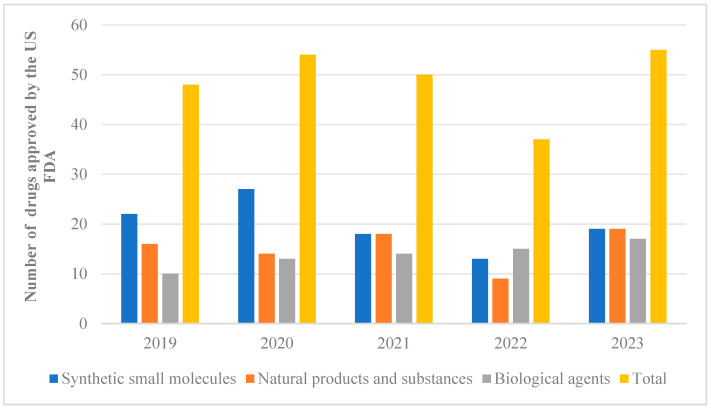
From 2019 to 2023, the US FDA approved 243 new drug classifications and statistics.

Natural products extracted from animals, plants, and microorganisms possess distinct structural characteristics and unique biological activities. Studies have demonstrated that these natural products can be utilized in the treatment of various diseases such as neurodegenerative disorders, cancer, and diabetes, among others [1,2,3,4]. These studies provide valuable references for the development and application of novel drugs. Out of the 2191 drugs and small-molecule new chemical entities approved for sale between 1981 and 2023, approximately 23.5 percent were directly or indirectly derived from natural products [5,6]. From 2019 to 2023, the US FDA approved a total of 243 new drugs, which were classified and counted. Among them, 76 drugs were developed based on natural pharmacophores (Figure 1). What is exciting is that a high proportion of natural products could be observed in approved synthetic drugs starting from 2019. However, there was a downward trend in 2022, followed by a significant increase in the number of natural products occurring in 2023. These findings suggest that natural products are regaining their appeal for the treatment of diseases. Notably, artemisinin [7], which was awarded the Nobel Prize in 2015, plays an extremely important role in combating parasitic diseases and holds significant value for the advancement of human medicine.

**Figure 2:**
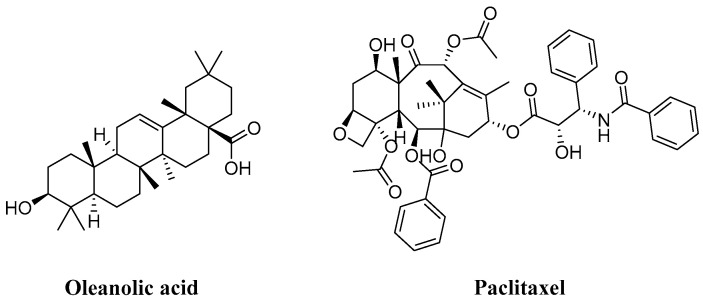
Chemical structure of oleanic acid and paclitaxel.

**Figure 3:**
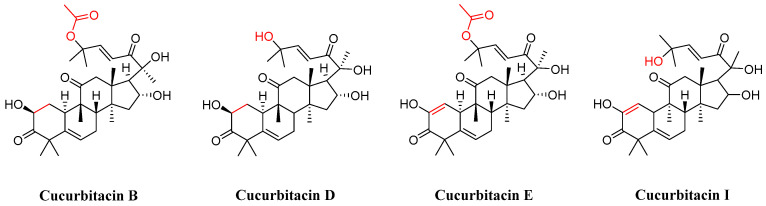
Chemical structure of cucurbitacin B, D, E, and I.

In the realm of natural products, triterpenoids are widely found in plants, exhibiting a variety of biological activities and possessing complex structures. Among them, tetracyclic triterpenoids and pentacyclic triterpenoids have been extensively studied [8]. Among pentacyclic triterpenes, oleanolic acid is particularly renowned for its excellent anti-inflammatory, antibacterial, antiviral properties [9,10]. Among tetracyclic triterpenes, paclitaxel stands out as the most famous one due to its significant anti-tumor activity and widespread use in cancer treatment (Figure 2) [11]. Cucurbitacins in the widely existing plant family also belong to tetracyclic triterpenoids, which are classified into 12 classes from cucurbitacin A to cucurbitacin T. Among these, cucurbitacins B and D appear more frequently, and researchers have analyzed and explored cucurbitacins B, D, E, as well as I (Figure 3). The key distinction among these four cucurbitacins lies in whether there is a double bond between C-1 and C-2 and whether C-25 is attached to a hydroxyl group or an acetyl group. Taking cucurbitacin B as an illustration, in cucurbitacin B, there is a single bond between C-1 and C-2, and C-25 is connected to an acetyl group. The difference is that in cucurbitacin D, C-25 is linked to a hydroxyl group, while in cucurbitacin E, there is a double bond between C-1 and C-2. Cucurbitacin I mainly features the connection of a hydroxyl group at position C-1 and C-2. Additionally, both cucurbitacins B and E inhibit the Wnt and STAT3 signaling pathways. Cucurbitacins B and I increase ROS levels. Cucurbitacin D inhibits STAT3 activation. Furthermore, cucurbitacins B, D, and E down-regulate the expression of key cell cycle regulators such as cyclin B1 [12]. Due to the high content of cucurbitacin B, significant pharmacological activity, and great development potential, numerous researchers have conducted research on it [13]. Cucurbitacin B is primarily found in cucurbitaceae and cruciferous plants. The fruit stalk of the Chinese medicine melon, which belongs to the cucurbitaceae family, is widely used in traditional Chinese medicine for treating abdominal distention and constipation due to its abundant presence of cucurbitin [14]. It can also be found in various other plants such as *Cucumis melo*, *Cucurbita andreana*, *Ecballium elaterium*, *Wilbrandia ebracteata*, and *Trichosanthes cucumerina*.

Since the isolation and extraction of cucurbitacin B, it has garnered extensive attention from researchers due to its high toxicity and narrow therapeutic window, necessitating structural modification and improvement in dosage form. Numerous derivatives have been designed and synthesized, with studies conducted on the effects of different dosage forms. From the past to the present, significant progress has been made in understanding its related molecular mechanism and target research. It has been reported that a research team used cucurbitacin B as a probe to directly target IGF2BP1 in liver cancer cells. These findings will provide valuable references for further development and research on cucurbitacin B [15]. Furthermore, considering the adverse effects of conventional pesticides and the beneficial impact of cucurbitacin B on various pests, the investigation into cucurbitacin B aims to offer valuable insights for the development of novel pesticides. According to the investigation and research, cucurbitacin B has been used as a listed drug in China, such as cucurbitacin tablets approved by the State Food and Drug Administration in 1998 for the adjuvant treatment of persistent hepatitis, chronic hepatitis, and primary liver cancer caused by dampness and heat toxicity. In China, there are 18 pharmaceutical companies that produce and sell cucurbitacin tablets or capsules for the treatment of chronic hepatitis and primary liver cancer. This paper reviews the biological and pharmacological research on cucurbitacin B and summarizes the latest progress on its structurally modified derivatives. The purpose of this study is to understand the therapeutic potential and value of cucurbitacin B as a drug discovery platform, aiming to provide valuable information for its development.

Cucurbitacin B exhibits a variety of pharmacological effects, including antioxidant and anti-inflammatory activities, protective effects on the nervous system, and excellent anti-tumor effects [16,17,18,19]. In this paper, based on numerous studies, the mechanism of action of cucurbitacin B in terms of antioxidant, anti-inflammatory, neuroprotective, cardiovascular, and anti-tumor properties, as well as its applications in agronomy, are introduced.

## 2. Pharmacological Activity

### 2.1. Antioxidant Activity

Oxidative stress is a negative effect produced by free radicals in the body and is thought to be an important factor in aging and disease [20]. Cucurbitacin B can reduce CCl_4_-induced oxidative stress in hepatocytes, and cucurbitacin B induces the intracellular accumulation of lipid ROS and the lipid peroxides MDA and Fe^2+^, while decreasing GSH levels [21,22].

Yang et al. [23] found that cucurbitacin B can also significantly reduce the mitochondrial volume and increase the mitochondrial membrane density, and cucurbitacin B can activate the SLC7A11 mitochondrial oxidative stress pathway to induce iron apoptosis, demonstrating its potential as a lead compound for the development of some anti-inflammatory drugs.

Previous studies have also shown that cucurbitacin B and cucurbitacin I have antioxidant properties, which can inhibit lipid peroxides [24]. In addition, Tehila et al. [25] found that cucurbitacin glycosides (cucurbitacin B and cucurbitacin E) have antioxidant and free radical scavenging capabilities, suggesting that cucurbitacins are expected to provide a preventive and therapeutic option for oxidation-damaged diseases.

### 2.2. Anti-Inflammatory Activity

Inflammation is a basic pathological process in which biological tissues are stimulated by certain stimuli such as trauma, infection, and other injury factors [26]. Inflammation can exacerbate the development of many complex diseases, such as diabetes, Alzheimer’s disease, cardiovascular disease, and age-related diseases such as cancer [27,28,29,30].

In recent years, many studies have shown that inflammation is closely related to the pathophysiological process of brain I/R injury. However, few studies have focused on the role of cucurbitacin B. Chu et al. [31] found that cucurbitacin B decreased OGD/R-induced cytotoxicity, ROS production, and levels of pro-inflammatory cytokines (including TNF-α, IL-1β, and IL-6) in OGD/R-induced cell damage models. These results suggest that cucurbitacin B can inhibit the activation of NLRP3 inflammasome to inhibit the inflammatory response and cell damage in brain I/R injury.

Sun et al. [18] found that cucurbitacins B, E, and I can significantly reduce the activation activity of NF-κB induced by TLR 2/4 agonists in cells. Pretreatment with these three elements can block the phosphorylation of JAK1, STAT1, and STAT3. At the same time, Nrf2/ARE signals can be up-regulated to protect cells from neuroinflammatory damage. Cucurbitacins B, E, and I can significantly inhibit neuroinflammatory response by inhibiting neuroinflammatory mediators in microglia stimulated by TLR 2/4 agonists.

Xue et al. [32] discovered that cucurbitacin B effectively inhibits the production of pro-inflammatory cytokines IL-1β, TNF-α, and IL-18 in ATP-stimulated macrophages. This inhibition is achieved by regulating TLR4 signaling to reduce the expression of NLRP3 inflammasome and suppress the generation of pro-IL-1β. Additionally, cucurbitacin B exhibits its inhibitory effects on NLRP3 activation and the subsequent formation of the NLRP3 inflammasome complex by downregulating ACS expression, thereby leading to a decrease in both IL-1β production and cleaved caspase-1 activity. Cucurbitacin B can also down-regulate key enzymes in the HIF-1α signaling pathway and glycolysis pathway. In addition, the data demonstrated that cucurbitacin B effectively mitigated MSU-induced arthritis in a murine model of gout. These findings suggest that cucurbitacin B functions as an inhibitor of the NLRP3 inflammasome, suppressing inflammation induced by various stimuli and exhibiting potential as an anti-inflammatory agent for diseases associated with the NLRP3 inflammasome.

Zhong et al. [33] conducted a study that demonstrated cucurbitacin B can reduce the influx of inflammatory cells and improve histology. Additionally, it regulated the expression of RANK/RANKL and OPG following the induction of periodontitis, and it decreased the levels of inflammatory mediators TNF-α and IL-1β in the gum tissue.

Liu et al. [34] validated cucurbitacin B as a possible anti-inflammatory component of Trichosanthis Pericarpium (TP)–Trichosanthis Radix (TR) through network pharmacology and intestinal microbiota sequencing.

Omar et al. [35] demonstrated experimentally that cucurbitacin B, the main component isolated from *Cucumis prophetarum*, exhibits the strongest anti-inflammatory activity, and they demonstrated its anti-inflammatory activity by slowing the elevated levels of TNF-a, IL-6, COX-2, and iNOS in carrageenan-induced prostates.

### 2.3. Neuroprotection

Mental illness, which accounts for 7 percent of China’s population, has overtaken heart disease and cancer to become the biggest burden on the country’s healthcare system, according to the World Health Organization [36]. Cucurbitacin B is worthy of further study for the treatment of such diseases. Ge et al. [37] discovered that a single administration of cucurbitacin B exhibited potential antidepressant effects in the forced swim test (FST) and tail suspension test (TST). Repeated administration of cucurbitacin B prevented depression-like behavior induced by chronic and unpredictable mild stress (CUMS). The repeated use of cucurbitacin B counteracted the downregulation of the hippocampal BDNF system caused by CUMS.

Li et al. [38] have demonstrated that cucurbitacin B potently promotes neurite outgrowth and lengthening in PC12 cells and primary neurons. Furthermore, the administration of cucurbitacin B has been shown to enhance the generation of new neurons in the hippocampus of ICR and APP/PS1 mice, thereby ameliorating the working memory deficits observed in these mice models. Notably, this compound does not appear to influence long-term memory. Collectively, these findings suggest that cucurbitacin B may represent a promising novel therapeutic candidate for the treatment of Alzheimer’s disease.

Liu et al. [39] demonstrated that cucurbitacin B mitigates the memory impairment induced by STZ-ICV in rats. The compound effectively reduced nitrous oxide stress in the STZ-ICV AD prototype brain, diminished the apoptosis of neuronal cells, downregulated the activity of acetylcholinesterase, and decreased the level of glutamate in the brain of STZ-ICV treated rats. Concurrently, cucurbitacin B enhanced the levels of GABA.

### 2.4. Cardiovascular Protection

Cardiovascular diseases encompass a spectrum of pathologies affecting the circulatory system, which can be categorized into acute and chronic conditions, predominantly associated with arteriosclerosis. Cardiovascular disease constitutes a significant proportion of annual mortality rates [40].

Xiao et al. [41] have demonstrated that cucurbitacin B exerts protective effects on the heart, including the prevention of hypertrophy, the amelioration of compromised cardiac function following myocardial infarction, the mitigation of fibrosis under pressure overload conditions, and the modulation of autophagic activity. This compound upregulated the expression of key autophagic markers such as LC3-II, Atg5, Atg7, Atg12, and Beclin1 while downregulating p62 expression. The study suggests that the cardioprotective mechanisms of cucurbitacin B may be mediated through the activation of autophagy, potentially via the inhibition of the Akt signaling pathway. Collectively, these findings indicate that cucurbitacin B may affect the autophagic process in cardiac tissue and cardiomyocytes, which may offer valuable insights for the therapeutic management of myocardial hypertrophy and heart failure.

Chen et al. [42] have demonstrated that cucurbitacin B exhibits a concentration-dependent inhibitory effect on lactate dehydrogenase (LDH) release induced by oxygen–glucose deprivation/reperfusion (OGD/R) in cardiomyocytes. Notably, cucurbitacin B potently reversed the inhibition of superoxide dismutase (SOD) products caused by OGD/R. Furthermore, this compound effectively attenuated apoptosis in cardiomyocytes subjected to OGD/R exposure. Importantly, cucurbitacin B has been shown to enhance cardiac function and promote fibrotic scar repair following ischemia/reperfusion (I/R) injury. Additionally, it significantly reduced the size of myocardial scars and enhanced fibrotic scar repair. These beneficial effects of cucurbitacin B are mediated through the JAK2/STAT3 signaling pathway, which not only facilitates cardiac function and myocardial repair following I/R injury but also mitigates LDH release, oxidative damage, and apoptosis in cardiomyocytes, thereby offering cardiomyocyte protection through modulation of the JAK2/STAT3 signaling pathway. Given the protective effect of cucurbitacin B on cardiac I/R injury, it provides a new route for the protection and treatment of I/R injury.

### 2.5. Antitumor Activity

**Figure 4:**
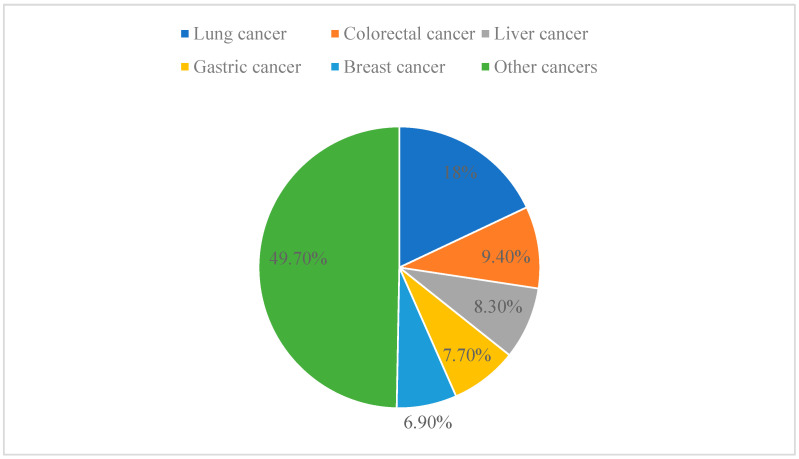
Different types of cancer as a proportion of total cancer.

Cancer is one of the leading causes of death worldwide and has even surpassed cardiovascular disease as the leading cause of premature death at this stage [43]. In 2020, the latest estimate from the World Health Organization (WHO) is that 9.95 million people will die of cancer worldwide. Among the different types of cancer, lung cancer is the leading cause of cancer death, accounting for 18.0% of all cancer deaths, followed by colorectal cancer (9.4%), liver cancer (8.3%), stomach cancer (7.7%), and breast cancer (6.9%) (Figure 4) [44]. Anticancer drugs aim to stop tumor growth by killing cancer cells and preventing tumor discovery and metastasis [45,46]. Traditional molecular sources are natural products that have a lot of potential in the field of pharmacology. The lead compounds of modern anticancer drugs are in part low-molecular-weight metabolites derived from terrestrial and marine organisms. Despite significant advances in cancer research over recent decades, anticancer therapy still faces serious challenges. Therefore, efforts have been made to explore new molecular mechanisms and develop new therapeutic strategies or drugs to treat cancer [47,48].

**Figure 5:**
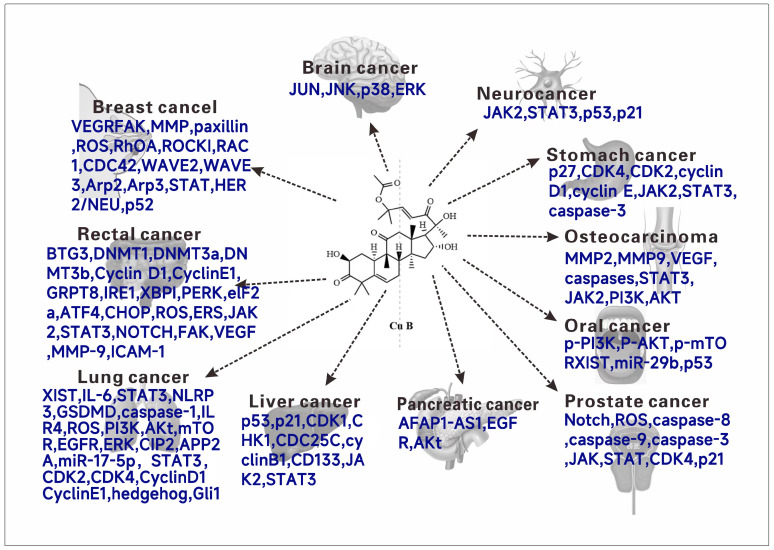
The anticancer molecular mechanism (Targets) of cucurbitacin B.

The natural product cucurbitacin B and its synthetic analogs have been studied and talked about, conducting experiments to explore cytotoxic activity against different human tumor cell lines. Various experiments and related reviews have also discussed or suggested possible mechanisms of action of cucurbitacin B. Cucurbitacin B has cytotoxic activity on a variety of tumor cells (Figure 5), and it has a good clinical application prospect as an antitumor drug. In this section, the antitumor activity of cucurbitacin B and its mechanism will be systematically introduced.

#### 2.5.1. Breast Cancer

Luo et al. [49] studied the effect of cucurbitacin B on MDA-MB-231 cells and found that cucurbitacin B could induce morphological changes in the cells, but it had no effect on their viability. It inhibited the migration and invasion of the cells, as well as cell adhesion to the matrix, type I collagen, fibronectin, and endothelial cells. Additionally, cucurbitacin B inhibited the phosphorylation of FAK and paxillin, and it could also induce the production of reactive oxygen species (ROS), which is helpful for the anti-metastatic potential of the cells.

Liang et al. [50] studied the effects of cucurbitine BMDA-MB-231 and SKBR-3 cells. Cucurbitacin B could significantly inhibit cell adhesion. It could decrease the deformability of cells. The expression levels of RAC1, CDC42, WAVE2/3, and ARP2/3 proteins in RhoA and ROCK1 and the RAC1/CDC42 pathways can be decreased, and the migration and invasion of cells in vitro can be inhibited.

#### 2.5.2. Glial Tumor

Yin et al. [51] studied the effects of cucurbitacin B on glioblastoma U87, T98G, U118, U343, and U373, and they found that cucurbitacin B inhibited the proliferation and clonal growth of glioblastoma. It can block the G2/M phase of the cell cycle, reduce cell migration, and induce cell apoptosis. Furthermore, it affects GBM cells through the JNK/c-Jun signaling pathway by increasing the levels of p-p38, p-JNK, and p-JUN.

#### 2.5.3. Neuroblastoma

Zheng et al. [52] studied the effect of cucurbitacin B on the neuroblastoma SH-SY5Y cell line. They found that cucurbitacin B induced early apoptosis in these cells and altered the expression of proteins involved in proliferation and apoptosis, such as up-regulating the p53 and p21 genes.

#### 2.5.4. Gastric Cancer

Xie et al. [53] studied the effect of cucurbitacin B on gastric cancer cells MKN-45 and found that cucurbitacin B inhibited the proliferation of these cells. It was observed that cucurbitacin B affected the cell cycle transition from the G0/G1 phase to the S phase. Additionally, cucurbitacin B upregulated the expression of p27 and downregulated the expression of CDK4, CDK2, cyclin D1, and cyclin E mRNA.

Xu et al. [54] studied the effects of cucurbitacin B on SGC7901, BGC823, MGC803, and MKN74 gastric cancer cells. Cucurbitacin B showed dose-dependent inhibition on gastric cancer cells. By inhibiting the STAT3 signaling pathway, it can decrease the phosphorylation of TYR-705 in STAT3 and inhibit the expression of the STAT3 target gene, thus inhibiting the growth and invasion of gastric cancer cells and inducing the apoptosis of cancer cells. The combination of cucurbitacin B and cisplatin (DDP) enhanced the activation of caspase-3 and the cleavage of caspase-3 substrate PARP, and it decreased the expression level of pSTAT3.

#### 2.5.5. Osteosarcoma

Zhang et al. [55] studied the effect of cucurbitacin B on human osteosarcoma cells (U-2OS). They found that cucurbitacin B could inhibit the proliferation of U-2OS cells. It significantly reduced the viability of U-2OS cells and affected the viability count of U-2OS cells. Additionally, cucurbitacin B induced apoptosis in U-2OS cells and inhibited the migration of these cells. It may down-regulate the expression of MMP2, MMP9, and VEGF, which could significantly inhibit cell migration and angiogenesis. Furthermore, cucurbitacin B promoted an increase in caspase levels and significantly inhibited the STAT3/JAK2 signaling pathway.

Wu et al. [56] studied the effects of cucurbitacin B on HOS and 143B cells. Cucurbitacin B inhibited the differentiation of M0 into M2 macrophages, resulting in a decrease in the number of M2 macrophages in vivo, and did not affect the polarization of M2-M1 macrophages. It can inhibit the differentiation of M2 macrophages by inhibiting the PI3K/AKT signaling pathway and then inhibiting the proliferation, migration, and invasion of osteosarcoma cells. The progression of osteosarcoma can be effectively slowed down by regulating the differentiation of M2 macrophages.

#### 2.5.6. Oral Squamous Cancer

Yu et al. [57] studied the effects of cucurbitacin B on CAL27 and SCC25 cells. They found that cucurbitacin B can regulate the expression of the PI3K-AKT pathway and reduce the expression levels of p-PI3K, p-AKT, and p-mTOR, thus inhibiting the migration of human tongue squamous cancer cells, blocking the cell cycle in the G2 phase, and inducing cell apoptosis, which inhibits the growth of oral squamous cell carcinoma.

Tao et al. [58] investigated the effects of cucurbitacin B on CAL27 and SCC9 cells. Cucurbitacin B plays an anticancer role by inhibiting the migration and invasion potential of oral squamous cell carcinoma cells. It can decrease the expression of XIST and increase the expression of miR-29b by inducing apoptosis and inhibiting cell growth through the p53 protein.

#### 2.5.7. Prostate Cancer

Ahmed et al. [59] studied the effect of cucurbitacin B on LNCaP cells and found that cucurbitacin B could inhibit the proliferation of these cells, alter their morphology, promote the activation of caspase, increase intracellular reactive oxygen species (ROS), and induce apoptosis; furthermore, the Notch signaling pathway in LNCaP cells was down-regulated.

Ahmed et al. [60] continued to study the effect of cucurbitacin B on PC-3 cells, and found that cucurbitacin B can reduce the activity of these cells. It promotes the activation of caspase-8, -9, and -3. Additionally, cucurbitacin B induces ROS-mediated oxidative stress, increases ROS levels, and triggers apoptosis. When acting on PC-3 cells, ROS can downregulate the expression of cyclin D1 and cyclin-dependent kinase 4 (CDK4) while enhancing the mRNA expression of the CDK inhibitor p21 Cip1. Furthermore, it significantly reduces the expression of the JAK/STAT signaling pathway.

#### 2.5.8. Pancreatic Cancer

Zhou et al. [61] studied the effects of cucurbitacin B on four types of cells: ASPC-1, BxPC-3, HPAC, and MiaPaCa-2. They found that cucurbitacin B can down-regulate the expression of AFAP1-AS1, thereby inhibiting cell proliferation. Additionally, by inhibiting the expression of AFAP1-AS1, it can block the cell cycle and inhibit the EGFR/Akt signaling pathway. Furthermore, cucurbitacin B regulates the mirNA-146B-5p/EGFR axis by modulating AFAP1-AS1, affecting cell proliferation.

#### 2.5.9. Hepatocellular Carcinoma

Li et al. [62] investigated the impact of cucurbitacin B on the growth of liver cancer cells, including the Huh7, Hep3B, and Hepa1/6 lines, demonstrating a significant inhibitory effect. Treatment with cucurbitacin B suppressed cell viability and proliferation, triggered the enrichment of differentially expressed proteins within the DNA damage repair pathway, and interrupted the cell cycle through the activation of DNA damage response. This activation led to the activation of the ATM-dependent p53-CDK1 and CHK1-CDC25C pathways, resulting in the increased expression of p53 and p21 and a concomitant decrease in cyclin-dependent kinase 1 (CDK1) levels. Additionally, cucurbitacin B enhanced the phosphorylation of checkpoint kinase 1 (p-CHK1) and inhibited the protein levels of cell division cycle 25C (CDC25C).

Wang et al. [63] investigated the impact of cucurbitacin B on HepG2 cells, revealing its broad anti-tumor properties in CD133+ HepG2 cells, including enhanced sensitivity to sorafenib. The synergistic combination of cucurbitacin B with sorafenib markedly suppressed the viability of CD133+ HepG2 cells. Additionally, cucurbitacin B inhibited the expression levels of cyclin B1, CDK1, and the JAK2/STAT3 signaling pathway in CD133+ HepG2 cells.

#### 2.5.10. Lung Cancer

Liu et al. [64] studied the effect of cucurbitacin B on A549 cells. Cucurbitacin B can inhibit the proliferation of lung cancer cells and promote apoptosis of lung cancer cells. It can inhibit the expression of XIST and the IL-6/STAT3 signaling pathway in cells, thus promoting the expression of miR-let-7c, and the overexpression of miR-let-7c can promote the regulation of lung cancer cells through the IL-6/STAT3 axis. In addition, the knockdown of XIST can enhance its influence on cell proliferation and apoptosis.

Yuan et al. [65] further explored the impact of cucurbitacin B on A549 cell behavior. Their findings indicate that cucurbitacin B effectively suppresses the morphological alterations, migration, and invasive capabilities triggered by TGF-β1 in these cells. Additionally, it inhibits the epithelial–mesenchymal transition (EMT) mediated by TGF-β1 and counteracts TGF-β1-induced EMT by suppressing reactive oxygen species production through the PI3K/Akt/mTOR signaling pathway.

Liu et al. [66] studied the effects of cucurbitacin B on human gefitinib-resistant NSCLC cell lines A549, NCI-H1299 (H1299), NCI-H1975 (H1975), and NCI-H820 (H820). Cucurbitacin B can inhibit the invasion and migration of gefitinib-resistant NSCLC cells and induce caspase-dependent apoptosis. It can induce lysosomal degradation of EGFR, thereby inhibiting the phosphorylation of ERK and Akt, and inhibit the CIP2A/PP2A signaling axis. Cisplatin (DDP) can enhance calucinin-B-dependent apoptosis and enhance its inhibition of the EGFR and CIP2A/Akt pathways.

Yu et al. [67] studied the effect of cucurbitacin B on PC9/GR cells and found that cucurbitacin B could improve the down-regulation of miR-17-5p expression induced by gefitinib, regulate the miR-17-5p/STAT3 axis, decrease the level of STAT3 protein, and inhibit phosphorylation. The suppression of cell proliferation and the enhancement of apoptosis conferred resistance to gefitinib in non-small cell lung cancer (NSCLC), a synergistic combination of cucurbitacin and gefitinib markedly potentiated apoptosis in PC9/GR cells.

Yu et al. [68] studied the effect of cucurbitacin B on A549/DDP cells and found that cucurbitacin B had a strong inhibitory effect on the proliferation of A549/DDP cells. It can block G0 and G1 phase cells, up-regulate the expression of p53 and cycle-related gene p21, and then inhibit the expressions of CDK2, CDK4, CyclinD1, and CyclinE1. In A549/DDP cells, the treatment significantly downregulates the expression levels of SMO and GLI1 RNA and proteins, which are pivotal molecules within the hedgehog signaling pathway.

#### 2.5.11. Rectal Cancer

Mao et al. [69] studied the effects of cucurbitacin B on SW480, Caco-2, HCT116, and Col205. Cucurbitacin B can reduce the levels of DNA methyltransferase (DNMT1, DNMT3a, and DNMT3b) through the demethylation of the BTG3 promoter in rectal cancer cells and re-activate BTG3.The cell cycle of cancer cells can be blocked in the G1 phase in vitro, and the levels of Cyclin D1 and Cyclin E1 in cancer cells can be significantly reduced.

Huang et al. [70] studied the effect of cucurbitin B on HT-29 and SW620 cells. Cucurbitacin B can inhibit the viability and proliferation of colorectal cancer cells. Furthermore, cucurbitacin B can increase the expression of GRP78 and activate the IRE1/XBP1 and PERK/eIF2α/ATF4/CHOP signaling pathways in UPR, demonstrating that it can produce ROS and induce ERS, which can induce apoptosis through ERS and its signal transduction pathway.

Zhang et al. [71] investigated the effect of cucurbitacin B on HCT116 cells and found that it inhibits the proliferation of colorectal cancer cells. Additionally, cucurbitacin B can inhibit the JAK2/STAT3 signaling pathway, reduce the migration and invasion ability of M2-like TAM polarization-induced colon cancer cells, enhance the anti-tumor response, and increase the expression of CD4 and CD8 in the tumor microenvironment.

Prasad et al. [72] studied the effects of cucurbitacin B on HCT116, SW480, and DLD1 cells and found that the combination of cucurbitacin B and cucurbitacin I could significantly inhibit the viability of the HCT116, SW480, and DLD1 cell lines. Both of them inhibit the G2/M cell cycle. They down-regulate the Notch signaling pathway and reduce the expression of CSC markers and the Notch signaling protein in tumor tissue.

#### 2.5.12. Bile Duct Cancer

Putthaporn et al. [73] studied the effect of cucurbitacin B on KKU-452 cells and found that cucurbitacin B significantly inhibited the activation of FAK and reduced the level of phosphorylated FAK protein. It could significantly decrease the amount of MMP-9 and inhibit the expression of ICAM-1 and VEGF. Cucurbitacin B can inhibit the migration, invasion, and adhesion of biliary duct cancer cells in a dose-dependent manner.

#### 2.5.13. Bladder Cancer

Yener et al. [74] explored the impact of cucurbitacin B on MB49 cell viability, documenting a reduction in cell proliferation in the presence of cucurbitacin B combined with cisplatin. Furthermore, cucurbitacin B induced apoptosis and autophagy within the tumor tissue of MB49 mice. The study also demonstrated that cucurbitacin B could enhance autophagy, as evidenced by increased levels of LC3II and Beclin-1 proteins, while concurrently decreasing the phosphorylation of p27, PRAS40, and raf-1. Additionally, the combination therapy attenuated the phosphorylation of AKT, ERK1/ERK2, mTOR, and BAD, while AMPKα levels were upregulated.

#### 2.5.14. Lymphoma

Mikinori et al. [75] studied the effect of cucurbitacin B on the primary invasive lymphoma cell lines BCBL-1, BC-1, GTO, and TY-1. Cucurbitacin B showed a dose-dependent effect on cell proliferation of PEL cell lines, which could be activated by caspase and then induce apoptosis. In addition, it can aggregate actin, inhibit p-cofilin levels, and then destroy cell shape, resulting in BBCL-1 cell stasis in the G2/M phase.

## 3. Research Progress of Cucurbitacin B Derivatives

The natural product exhibits good biological activity but also has certain limitations, necessitating structural modifications to enhance its therapeutic effect and overcome these limitations in order to ultimately develop a safe and more effective drug. For cucurbitacin B, it was first isolated from the bitter principle of cucurbitaceae in 1957 [76,77]. Until now, no studies on the total synthesis of cucurbitacin B have been found. The most recent study is that of Michae et al. [78], focusing on the synthesis of the cucurbitacin core by the Diels–Alder reaction, which has a guiding effect on the total synthesis of cucurbitacin B.

The high toxicity of cucurbitacin B poses a significant challenge that necessitates immediate resolution, rendering it an urgent concern in drug development. Therefore, it is imperative to design and synthesize a series of derivatives based on cucurbitacin B as a lead compound in order to elucidate its structure–activity relationship and identify compounds with reduced toxicity and enhanced anticancer efficacy. Presented herein is a review highlighting recent advancements in the synthesis of novel cucurbitacin B derivatives.

### 3.1. Modification and Pharmacological Activity of 2-Hydroxyl Group

**Figure 6:**
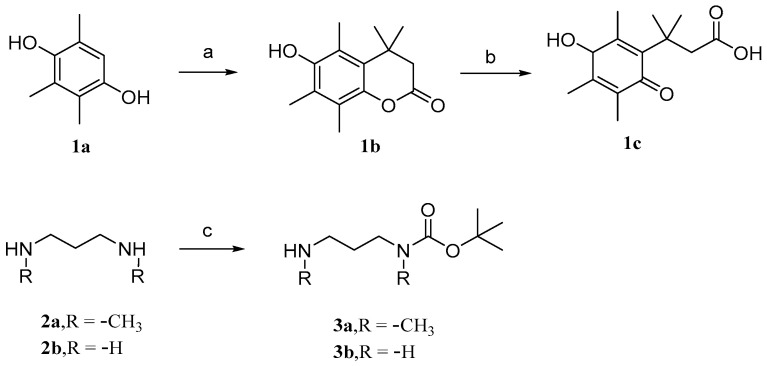
Synthetic routes of compounds **1a**–**1c**, **2a**–**2b,** and **3a**–**3b**. Reagents and conditions: (a) 3,3-dimethylacrylic acid, CH_3_SO_3_H, 70 °C; (b) NBS, acetone, H_2_O; (c) Boc_2_O, DCM.

**Figure 7:**
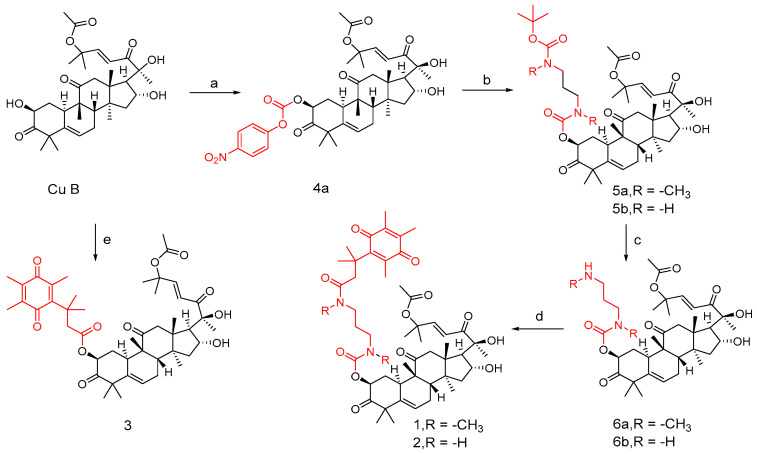
Synthetic routes of compounds **1**, **2**, and **3**. Reagents and conditions: (a) 4-nitrophenyl chloroformate, Et_3_N, DCM, 0 °C; (b) compounds 3a and 3b, Et_3_N, DCM; (c) 10%TFA, DCM; (d) compound 1c, EDCI, DMAP, DCM; (e) 1c, EDCI, DMAP, DCM.

Parichat et al. [79] designed a prodrug based on cucurbitacin B to reduce its toxicity to normal cells while maintaining or enhancing its anticancer activity. Figure 6 and Figure 7 depict the synthetic pathways for compounds **1**, **2**, and **3**. Firstly, trimethylhydroquinone reacts with 3,3-dimethacrylic acid in the presence of anhydrous methanesulfonic acid at a controlled temperature of 70 °C. Then, it is treated with *N*-bromosuccinimide (NBS) in an acetone aqueous solution to obtain compound **1c**. In DCM, the addition of dibutyl dicarbonate (Boc_2_O) to 1,3-diaminopropane produces monoBOC-protected intermediates **3a** and **3b**.

In DCM, cucurbitacin B was added to triethylamine and 4-nitrophenylchloroformate at 0 °C to obtain compound **4a**. Similarly, compounds **3a** and **3b** were obtained by adding triethylamine to cucurbitacin B. To obtain intermediates **5a** and **5b**, add them in the same way as before. Add 10% TFA to solutions of compounds **5a** and **5b** in DCM medium to obtain intermediates **6a** and **6b**. Compounds **1** and **2** can be obtained by adding compound **1c**, EDC, and DMAP to intermediates **6a** and **6b**, respectively. Similarly, compound **3** can be obtained by adding the mixed system to cucurbitacin B.

To evaluate the cytotoxicity of cucurbitacin B and compounds **1**, **2**, and **3** against breast cancer (MCF-7) and non-cancer Vero (African green monkey kidney) cells. Tamoxifen (TAM), a known anticancer drug, was used as a positive control (Table 1). Cucurbitacin B itself exhibited good activity against MCF-7 cells (IC_50_ = 12.0 µM), while compounds 1, 2, and 3 demonstrated IC_50_ values of 18.1, 15.4, and 16.6 µM, respectively, indicating better cytotoxicity against MCF-7 cells compared to TAM. Cucurbitine B exhibited high toxicity towards non-cancerous Vero cells (IC_50_ = 0.04 µM), while compounds **1**, **2**, and **3** showed low cytotoxic activity against Vero cells, being 310, 47, and 2 times less toxic than cucurbitine B, respectively [79]. To some extent, the introduction of quinone fragments at the 2-hydroxyl group of cucurbitacin B adversely affects its anti-tumor activity.

**Figure 8:**
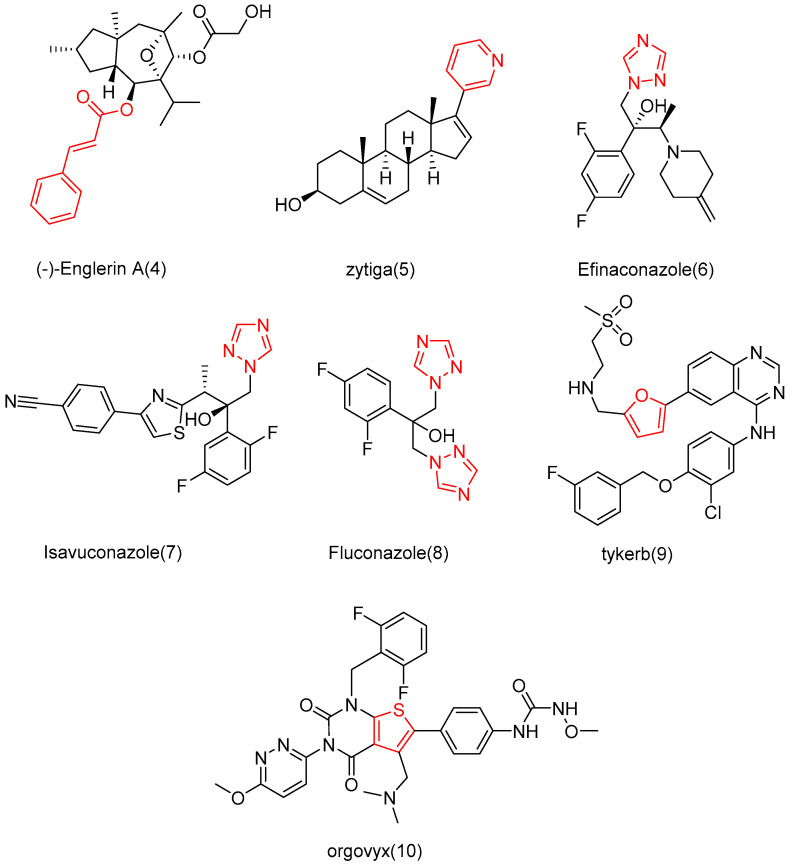
Chemical structure of marketed drugs.

Drugs are often designed and synthesized using cinnamic acid, pyridine/furan, and triazolyl [80,81,82,83]. For example, cinnamic acid is found in many active molecules with good efficacy, such as (−)-Englerin A (**4**) and zytiga (**5**), which contains the pyridine part, and has been approved for marketing by the FDA. In addition, triazole has been successfully applied in the development of Efinaconazole (**6**), Isavuconazole (**7**), Fluconazole (**8**), Tykerb (**9**), and Orgovyx (**10**), which contain furan or thiophene fragments (Figure 8). Since these fragments are commonly used in drug research, Shang et al. [84] utilized them to modify the 2-site OH of cucurbitacin B with the hope of enhancing anti-tumor activity and reducing cytotoxicity.

**Figure 9:**
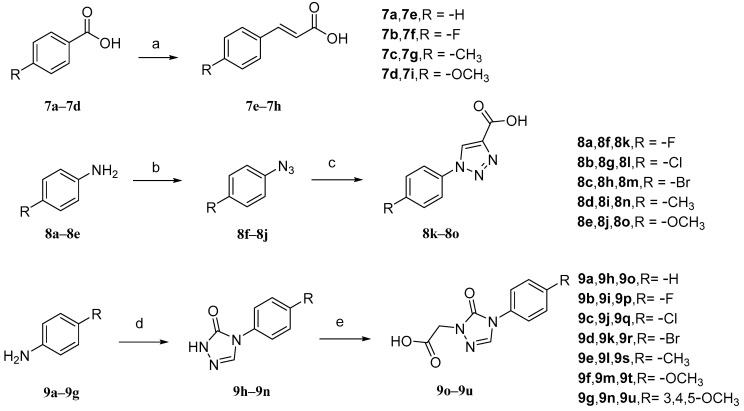
Synthetic routes of intermediates **7e**–**7h**, **8k**–**8o**, and **9o**–**9u**. Reagents and conditions: (a) malonic acid, pyridine, DMF, 90 °C, 6 h; (b) (i) 10% HCl, NaNO_2_, H_2_O, 0–5 °C, 30 min; (ii) NaN_3_, H_2_O, 0–5 °C, 2–4 h; (c) propiolic acid, L-ascorbic acid sodium salt, CuSO_4_·5H_2_O, *n*-BuOH/H2O, r.t., 24 h; (d) triethyl orthoacetate, methyl carbazate, methanol, 78 °C, reflux, CH_3_ONa; (e) chloroacetic acid, CH_3_CN, K_2_CO_3_, 60 °C.

The intermediates **7e**–**7h** (Figure 9) were initially prepared. Malonic acid and pyridine were added to benzaldehyde (**7a**–**7d**) with different *para*-substituents, and the solvent used was DMF. The reaction temperature was maintained at 90 °C. Intermediates **8k**–**8o**, aniline with different *para*-substituents (**8a**–**8e**), NaNO_2_, 10% hydrochloric acid, and solvent (H_2_O) were used. Sodium azide was added after the reaction for 30 min to obtain intermediates **8f**–**8j**. Then, sodium ascorbate, propionic acid, and copper sulfate pentahydrate were added. The solvents that were used were *n*-BuOH and H_2_O. Intermediates **9o**–**9u**, aniline with various substituents (**9a**–**9g**), triethyl orthoacetate, methyl hydrazyl formate, and sodium methanol were added to MeOH to form **9h**–**9n**. Then, chloroacetic acid and K_2_CO_3_ were added to react with **9h**–**9n** in acetonitrile at a controlled temperature of 60 °C.

**Figure 10:**
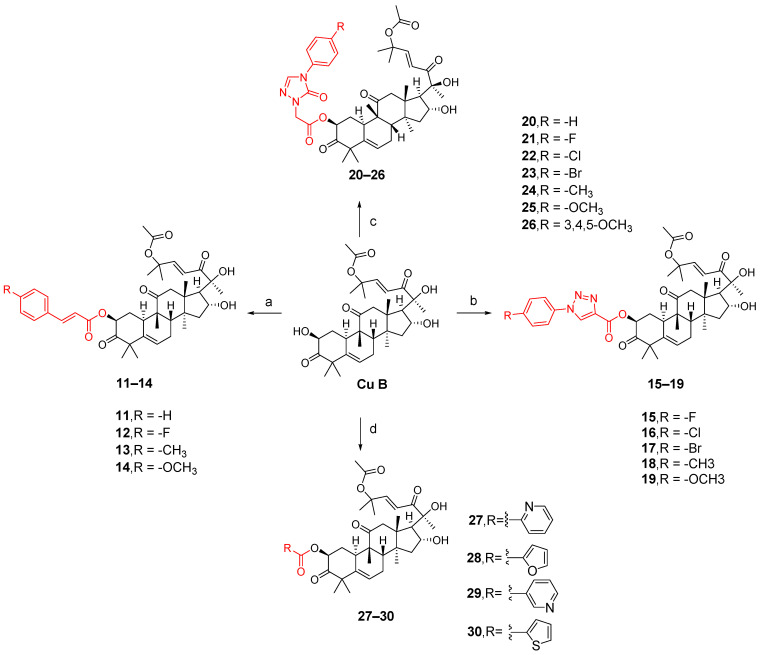
Synthetic routes of compounds **11**–**30**. Reagents and conditions: (a) 1,2-dichloroethane, **7e**–**7h**, EDCI, DMAP, 37 °C, 16 h; (b) 1,2-dichloroethane, **8k**–**8o**, EDCI, DMAP, 60 °C, 20 h; (c) 1,2-dichloroethane, **9o**–**9u**, EDCI, DMAP, 80 °C, 24 h; (d) 1,2-dichloroethane, 0.5 equiv 2-picolinic acid, 2-furoic acid, nicotinic acid or 2-thiophene carboxylic acid, EDCI, DMAP, 65 °C, 18 h.

The synthesis of compounds **11**–**14** involved the addition of 1,2-dichloroethane (DCE), EDCI, DMAP, and cucurbitacin B to intermediates **7e**–**7h** for reaction at a temperature of 37 °C. Compounds **15**–**19** were synthesized by adding EDCI, DMAP, cucurbitacin B, and intermediates **8k**–**8o** to DCE at a temperature of 60 °C. For compounds **20**–**26**, all other conditions remained the same except that the temperature was controlled at 80 °C. Compounds **27**–**30** involved reacting four carboxylic acid intermediates (2-pyridinic acid, 2-furanoic acid, niacin or 2-thiophenic carboxylic acid) with cucurbitinol B. All other conditions remained the same, but the reaction took place at a temperature of 65 °C (Figure 10). Except for compound **18**, compounds **15** and **20**–**27** exhibited comparable or slightly reduced anti-proliferative activity against HCT-116 (Table 2). Furthermore, these compounds demonstrated a weak inhibitory effect on the growth of normal L02 cells, indicating their good safety profile. Compound **21** exhibited the highest selectivity index for anti-proliferative activity, with an SI value of 2.94, which was five times higher than that of cucurbitacin B. To further investigate the anticancer potential of compound **21**, ten different cancer cell lines, including A549, HCT116, MDA-MB-231, MCF-7, SK-OV-3, HeLa, HepG-2, BXPC-3,PANC-1, and CFPAC-1, were tested. As shown in Table 3, compound **21** demonstrated comparable or slightly improved anticancer activity compared to cucurbitacin B while exhibiting superior tumor specificity. Among these cancer cells, A549 was the most sensitive to compound **21**, with an IC_50_ value of 0.009 μM and an SI value of 11.11, which was superior to cucurbitacin B, suggesting that compound **21** has potential for cancer treatment [84].

The structure–activity relationship of this series of compounds demonstrates that the activity of the 1,2,4-triazolone fragment introduced into the 2-hydroxyl group of cucurbitacin B is superior to that of cinnamic acid, 1,2,3-triazole, and other five-membered heterocyclic fragments. Furthermore, among compounds **20**–**26**, the preliminary structure–activity relationship indicates that the introduction of stronger electron-withdrawing groups in the *para*-position of the phenyl group or more electron-donating groups on the phenyl group is advantageous for inhibiting the proliferation of HCT116.

### 3.2. Modification and Pharmacological Activity of 16-Hydroxyl Group

**Figure 11:**
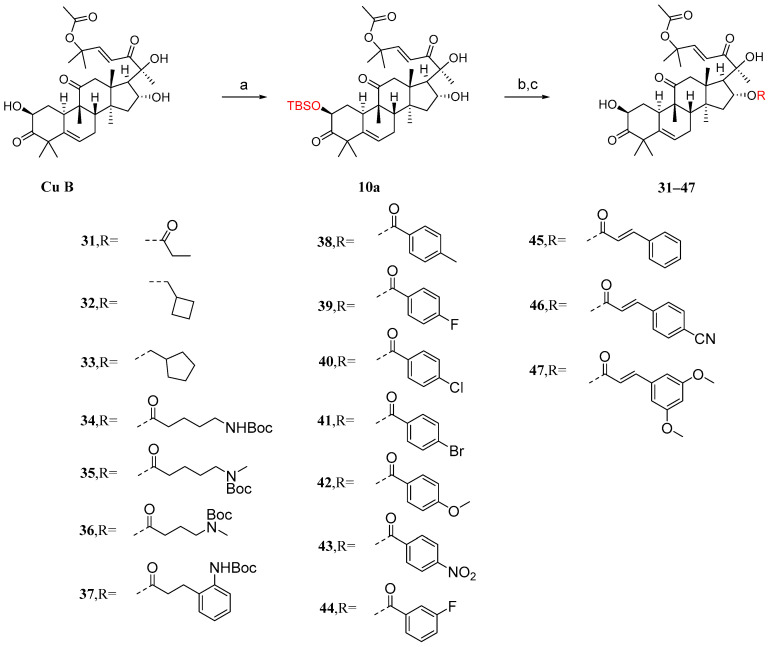
Synthetic routes of compounds **31**–**47**. Reagents and conditions: (a) TBSCl, imidazole, DCM; (b) RCOOH, EDCI, TEA, DMAP, DCM; (c) TBAF/AcOH, THF.

It has been reported that the introduction of an acyl group in the 2-hydroxyl moiety can significantly reduce anticancer activity [85]. Ge et al. [86] introduced acyl groups into the 16-hydroxyl segment to investigate their impact on anticancer activity. Due to the higher reactivity of the 2-hydroxyl group compared to the 16-hydroxyl group, it is easier to introduce acyl groups there. Therefore, protection of the 2-hydroxyl group is necessary. As shown in Figure 11, in the presence of imidazole, cucurbitacin B was treated with T-butyldimethylsilyl chloride (TBSCl) to obtain the TBS-protected intermediate **10a**. Since the hydroxyl group of C-20 is a tertiary hydroxyl group, the steric hindrance at C-20 is higher than that at C-16, making the esterification reaction difficult. Intermediate **10a** was added to a dichloromethane solution along with EDCI, DMAP, and TEA to reverse the esterification reaction using different carboxylic acids. Then, in a THF solution, TBAF or acetic acid was added to remove the TBS protection. This resulted in compounds **31**–**47** with yields ranging from 48% to 86%.

**Figure 12:**
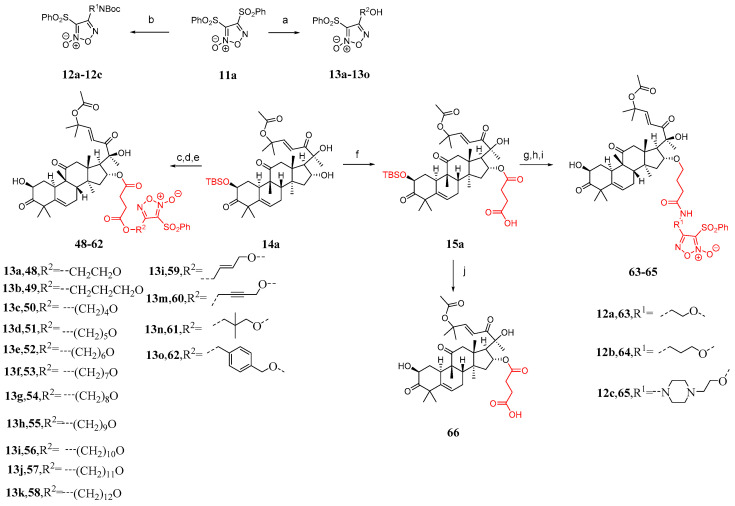
Synthetic routes of compounds **48**–**66**. Reagents and conditions: (a) corresponding alcohol amine, NaOH, THF; (b) corresponding diol, NaOH, THF; (c) **13a**–**13o**, succinic anhydride, DMAP, DCM; (d) EDCI, TEA, DMAP, DCM; (e) TBAF, AcOH, THF; (f) succinic anhydride, DMAP, DCM; (g) **12a**–**12c**, TFA, DCM; (h) HATU, DIPEA, DMF; (i) TBAF, AcOH, THF; (j) TBAF, AcOH, THF.

Some studies have shown that the toxicity of bruceopitrol can be reduced by introducing a phenylsulfonyl-substituted furacil NO release fragment [87,88,89,90,91]. Therefore, Ge et al. [86] designed 18 cucurbitacin B derivatives (Figure 12) and introduced phenylsulfonyl-substituted furapyridine NO-releasing fragments. Compound **11a** reacted with different diols or alkanols to yield **12a**–**12c** or **13a**–**13o**, respectively. The desired carboxylic acid was synthesized by adding DMAP and succinic anhydride to compounds **13a**–**13o**, followed by esterification with compound **14a** and removal of the TBS group, resulting in the formation of compounds **48**–**62** in two steps. Compound **15a** was obtained from the reaction between compound **14a** and succinic anhydride. Compounds **2a**–**2c** were esterified with compounds **12a**–**12c** by removing the Boc group using TFA, followed by the addition of HATU and DIPEA and the removal of the TBS group to yield compounds **63**–**65**. Compound **66** was obtained by directly removing the TBS group from compound **15a**.

The activity against the proliferation of the human hepatocellular carcinoma cell line HepG2 and the normal hepatocellular cell line L02 was evaluated [85]. Cucurbitacin B exhibits good activity against HepG2 cells, but its high toxicity results in a low therapeutic index (TI). The compounds in the **31**–**47** subseries were found to be less cytotoxic to L02 cells compared to cucurbitacin B, but their antihepatoma activity against HepG2 cells was also significantly reduced. Among the compounds in the **31**–**47** subseries, compound **45** exhibited the highest TI value (Table 4). Furthermore, the introduction of electron-withdrawing groups in the phenyl *para*-position of cinnamic acid appears to be beneficial against HepG2 proliferation; however, it simultaneously increases cytotoxicity towards L02 cells.

The compounds **48**–**62** exhibited effective activity against HepG2 cells, with a significant decrease in their cytotoxicity against L02 compared to cucurbitacin B, as shown in Table 5. Compounds **63**–**65** showed lower toxicity compared to cucurbitacin B, resulting in a slight increase in TI values. Compound **49** demonstrated a significantly higher TI value, showing a 14.7-fold increase compared to cucurbitacin B, suggesting its potential as a treatment for liver cancer [86]. The structure–activity relationship analysis of compounds **48**–**58** suggests that a carbon chain length ranging from 3 to 5 is optimal.

### 3.3. Modification and Pharmacological Activity of C-25 Acetyl Group

**Figure 13:**
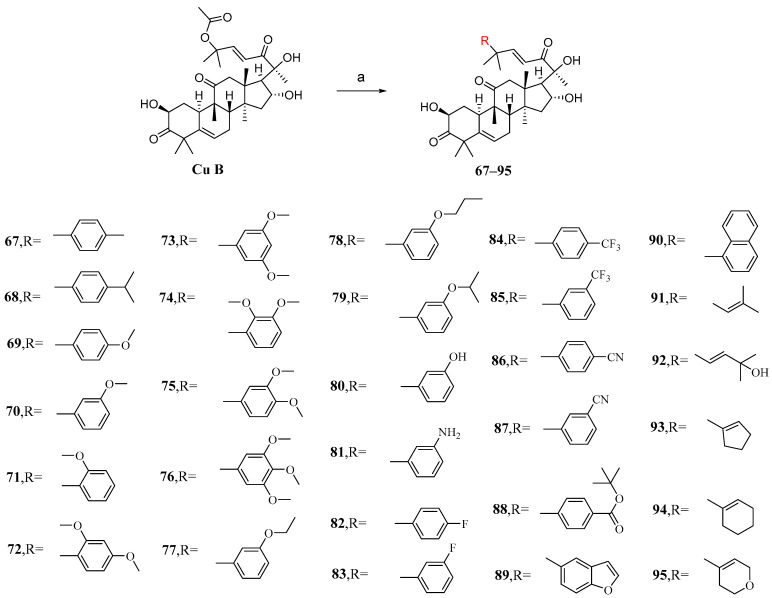
Synthetic routes of compounds **67**–**95**. Reagents and conditions: (a) RB(OH)_2_, KF, Pd_2_(dba)_3_, DCM, rt.

The main purpose of modifying C-25 acetoxy in Zhuo et al.’s study was to enhance the stability and pharmacokinetic properties of cucurbitacin B while maintaining or improving its antiproliferative activity [92]. After conducting numerous experiments to determine the optimal conditions, it was found that Pd_2_(dba)_3_ exhibited the highest catalytic yield as a palladium catalyst. Cucurbitacin B was introduced into a DCM solution containing KF and Pd_2_(dba)_3_ to react with various intermediates, resulting in the production of compounds **67**–**95** (Figure 13).

The synthesized compounds **67**–**95** were all evaluated in vitro for their cytotoxic activity against A549 cells. Compound **87**, with an IC_50_ value of 6.8 nM, exhibited stronger cytotoxicity than cucurbitacin B, being twice as potent (Table 6) [92]. The structure–activity relationship reveals that the introduction of substituents with reduced steric hindrance at the *meta*-position of the phenyl group enhances antiproliferative activity.

### 3.4. Co-Modification of 2-OH and 16-OH

**Figure 14:**
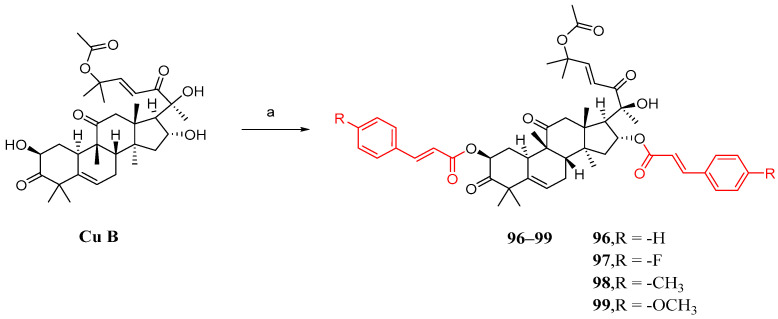
Synthetic routes of compounds **96**–**99**. Reagents and conditions: (a) 1,2-dichloroethane, **7e**–**7h**, EDCI, DMAP, 37 °C, 16 h.

Shang et al. synthesized cucurbitacin B derivatives using the same method as described previously. However, the results indicated that the simultaneous introduction of substituted cinnamic acid fragments on both the 2 and 16 hydroxyl groups had a detrimental effect on anti-NSCLC activity (Figure 14) [84].

### 3.5. Study on Modification and Pharmacological Activity of Other Sites

**Figure 15:**
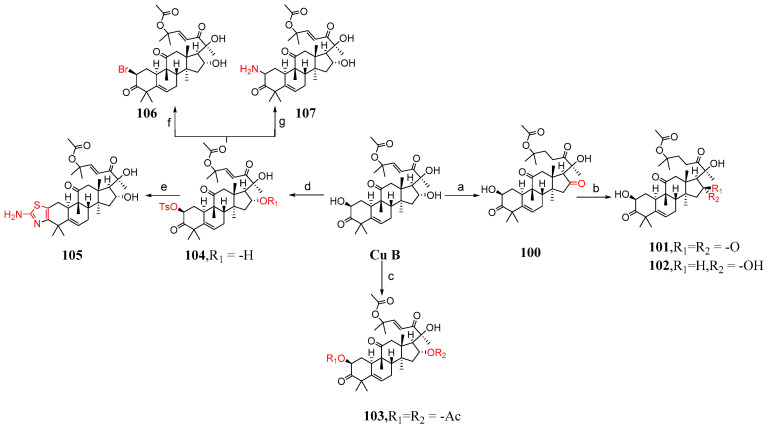
Synthetic routes of compounds **100**–**107**. Reagents and conditions: (a) PCC, BaCO_3_, CH_2_Cl_2_; (b) KOH, MeOH, DMF; (c) uccinic anhydride, Py, DMAP; (d) 4-toluenesulfonyl chloride, DABCO, CH_2_Cl_2_, 0 °C; (CH_3_CO)_2_O, Py, DMAP; (e) SC(NH_2_)_2_, EtOH, MW, 100 °C; (f) (Bu)_4_N^−^Br+, DMF; (g) NaN_3_, DMF, 70 °C.

**Figure 16:**
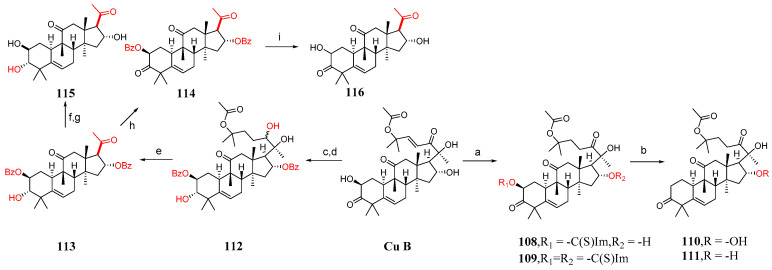
Synthetic routes of compounds **108**–**116**. Reagents and conditions: (a) 1,1′-thiocarbonyldiimidazol, Et_2_Cl_2_, 60 °C; (b) [CH_3_(CH_2_)_10_CO]_2_O_2_, Ph_2_SiH_2_, toluene, 115 °C; (c) C_6_H_5_COCl, Py, CH_2_Cl_2_; (d) C_12_H_28_AlO_3_Li, THF, 0 °C; (e) H_5_IO_6_, MeOH; (f) PCC, BaCO_3_, CH_2_Cl_2_; (g) ethylene glycol, HC(OEt)_3_, TsOH; (h) PCC, BaCO_3_, CH_2_Cl_2_; (i) ethylene glycol, HC(OEt)_3_, TsOH, KOH, MeOH, HCl, Et_2_O.

This section mainly describes C-2 and C-16 modification, A-ring modification, deoxygenation reactions, and side chain cleavage. Karen et al. [85] synthesized compounds **100**–**116** by modifying cucurbitacin B (Figure 15 and Figure 16). They added BaCO_3_ to cucurbitacin B and PCC and then synthesized compound **100**. Compound **100** was treated with KOH in a mixed solvent of MeOH and DMF to form compounds **101** and **102**. The hydroxyl group of cucurbitacin B was protected by reacting it with acetic anhydride and cucurbitacin B, resulting in the formation of **103**. By adding 4-toluene sulfonyl chloride to DCM, DABCO reacted with cucurbitacin B at a low temperature to produce **104** or under microwave irradiation at 100 °C to yield **105**. Compound **104** underwent a reaction with (Bu)_4_NBr in DMF, generating compound **106**, or with sodium azide in DMF, producing compound **107**. Cucurbitacin B reacted with 1,1′-thiocarbonyl diimidazole and dichloroethane at 60 °C to produce compounds **108** and **109**. Compounds **110** and **111** were synthesized by treating compounds **108** and **109** with diphenylsilane and peroxide lauryl alcohol in refluxing toluene. Cucurbitacin B was reacted with benzoyl chloride and pyridine in DCM, followed by reduction using tert-butyl-lithium aluminum hydride to obtain compound **112**. Compound **112** was further treated with periodic acid in MeOH to yield compound **113**. Subsequently, compound **113** was subjected to a reaction with triethyl orthoformate in ethylene glycol, followed by *p*-toluene sulfonate addition, KOH hydrolysis, and subsequent HCl treatment to afford compound **115**. Additionally, compound **113** underwent a reaction with PCC and BaCO_3_ in DCM, resulting in the formation of compound **114,** which could be obtained through the synthesis step leading to compound **115**.

Compounds **104**, **107**, **108**, and **110** demonstrated moderate activity against A549 tumor cells. Notably, compounds **107** and **108** were 100 times more potent than cucurbitacin B (Table 7). To some extent, substituting the 2-hydroxyl group of cucurbitacin B with different bioisosteres might potentially result in improved anti-tumor compounds.

## 4. Studies on Cucurbitacin B Preparations

Based on the unique activity of natural products, more and more natural products have been discovered and applied, and any drug must be prepared into a suitable application form before clinical use in order to solve the problems of achieving the best therapeutic effect of drugs, reducing adverse reactions, making different patients more convenient to use and accept, facilitating the carrying and transportation of drugs, etc. More and more new drug preparations appear in the public field of vision, such as microemulsions, solid liposomes, micelles, and so on [93,94,95].

Tian et al. [96] studied cucurbitacin B microemulsions (CuB-ME). An o/w CuB ME was prepared, and the CUP-ME formula was optimized, with azone:Tween 80:ethanol:water = 2.5:16.9:5.6:75.0 (*w*/*w*), where the nitrogen ketone can increase the solubility of CuB and promote the amount of skin permeability. While the water affects the particle size and permeability coefficient of CUP-ME, hydration can also promote the penetration of drugs. In addition, pharmacodynamic studies and irritant test results showed efficacy and safety. In conclusion, CuB-ME in this study can improve patient compliance and characterize the skin drug delivery process promoted by CUB-ME at the molecular level, which provides a reference value for the application of cucurbitacin B in the transdermal drug delivery system.

Wu et al. [97] prepared CuB-SD with a weight ratio of 1:7 by following the solvent method using PLX-407 as the carrier. After the dissolution test and pharmacokinetic study, it can be proved that solid dispersion technology can significantly improve the solubility and oral bioavailability of CuB, which can improve patient compliance.

Lv et al. [98], using carboxymethyl chitosan (CCS) as a bioadhesive polymer, glycerin as a plasticizer, and phospholipid–bile salt mixed micelles (PL-BS-MMs) as nanoscale carriers, developed an oral delivery system for muco-adhesive cucurbitacin B. After optimization, it has good physical properties and release characteristics in vitro. It can be proved by oral administration experiments in rabbits that a nano-scale chitosan film preparation can replace the oral dosage form because of its good pharmacokinetic characteristics. This oral mucosal adhesive membrane may provide an alternative route for the safe delivery of cucurbitacin B with better patient compliance and higher bioavailability.

Hu et al. [99] prepared solid lipid nanoparticles (Cu-BSLNs) loaded with cucurbitacin B by emulsification ultrasound. It was found that Cu-B SLNs can passively target tumors with the EPR effect and show higher accumulation in tumor interstitial space, which can improve the efficacy of cucurbitacin B and reduce the dose. This suggests that SLNs can increase the passive targeted delivery of drugs and reduce the dose and toxicity, which can improve the efficacy of insoluble drugs.

Chen et al. [100] conjured two cucurbitacin B molecules with diselenide bonds to form a dimeric prodrug, which was then assembled with FA-PEG-DSPE to finally produce an FA-Se-Se-NP nanomedicine. Diselenide bonds and CuB work synergistically to amplify oxidative stress and kill cancer triple-negative (TNBC) cells. FA-Se-Se-NPs have a longer blood circulation time and can also selectively accumulate in tumor tissue through passive targeting mediated by EPR effects. In an environment of high intracellular ROS and GSH levels, diselenide bonds can break and consume GSH, produce ROS, release CuB, and then produce ROS, amplify intracellular oxidative stress, and kill cancer cells. This new approach provides a promising strategy for cancer therapy, with significant effects on TNBC in vivo and in vitro.

Tang et al. [101] used ion exchange resins to simply isolate collagen cationic CPs from bovine collagen peptide (CPs) to modify surface mixed nanomicelles (MM) and obtained CPS-modified CuB mixed nanomicelles (CUB-MS-CPS). In vitro, CPS-modified nanomicelles can lead to a significant increase in cell uptake and transport. Cub-ms-cps can enhance the oral absorption of CuB. Therefore, the use of CPs as a carrier may provide an effective method for oral drug delivery, which may help to address the problem of drug diffusion and penetration barriers in the intestine.

## 5. Research Progress on Cucurbitacin B in Agronomy

Nowadays, the existence of pests has made the economic situation of agriculture worse, and traditional synthetic chemical insecticides are still used to control pests, but these pesticides have caused damage to human health and the ecological environment, in addition to causing pest resistance [102,103,104]. CuB is the main secondary metabolite of the host plant calabash. It is easily extracted and isolated from plants such as calabash and is toxic to some arthropods [105,106,107,108]. A previous study showed that high concentrations of CuB can increase aphid mortality and decrease their fitness [109]. Therefore, we need to reduce the use of chemical pesticides and provide new ideas for the synthesis of novel biopesticides.

The survival and reproduction of animals need the help of taste. Grs is the first taste receptor found in the genome of fruit flies [110]. Suman et al. [111] conducted an experiment of the genetic screening method for electrophysiological examination, and the results show that flies have food resistance to Cucc-B, which is mediated by GR33a, and Cucc-B has an insecticidal effect. It can be proved that GR33a is the molecular sensor required for Cucc-B taste detection (CUCC-B). Because Cucc-B is widely found in cucurbitaceae plants and because of the specific GRs, the development of new highly effective insecticides based on cucurbitacin B is of paramount importance.

Sterols play an important role in cell membrane homeostasis and also affect steroid production in animals. Insects lack the ability to synthesize sterols, so they need sterol supplements for growth and development [112,113]. Insects can synthesize the steroid hormone ecdysone from dietary cholesterol in the steroid-producing organ, the prethymus (PG) [114]. Miwako et al. [115] investigated the effects of cucurbitacin B on the development of Drosophila melanogaster. The experimental results showed that CuB could prevent the physiological process of stunting their development. CuB destroyed any function related to sterols, and ecdysone biosynthesis in PG was inhibited. CuB can be used as an inhibitor of ecdysone biosynthesis and an antagonist of ecdysone receptors. In addition, a steroid endemic to arthropods is ecdysone, the biosynthetic pathway is an ideal target for IGRs, and CuB may serve as a candidate molecule for the development of a novel IGR that inhibits ecdysone biosynthesis.

The health of plants is closely linked to the subsurface microbial ecosystem [116]. Several studies have shown that plant-specific metabolites play an important role in the formation of the rhizosphere microbiota [117]. Zhong et al. [118] found that the transport of cucurbitacin B from melon roots to soil can selectively enrich two types of bacteria (Enterobacter and Bacillus) to regulate the rhizosphere microbiota, which in turn can produce strong resistance to the fusarium pathogen. The coordinated regulation of cucurbitacin biosynthesis and transport may be a potential mechanism to balance the pathogen resistance induced by cucurbitacin and reduce excessive energy depletion in plants.

Cotton aphids, which are distributed throughout the world, pose serious economic and ecological problems due to their rapid reproduction and high resistance to pesticides [103]. The development of insecticide resistance is rapid and widespread, threatening crop productivity. In contrast, biopesticides (e.g., fungi and plant extracts) thus become a better pest control option [119,120]. Zhao et al. [121] investigated the effects of cucurbitacin B on two host biotypes, cotton- and cucurbit-specialized aphids (CO and CU), and showed that cucurbitacin B significantly reduces fitness at the population level of cotton aphids and can alter important detoxification enzyme activity. Because of this property, CuB may have the opportunity to be developed as a pesticide for aphid control in agriculture, and it is also of great significance for the control and protection of cotton aphids and other insect pests.

Laurence et al. [122] experimentally demonstrated that cucurbitacin B prevents 20-hydroxyecdysone (20E) stimulation of the hormone reactivity reporter gene and also prevents the formation of the drosophila epidermal steroid receptor Ultraspiracle 20E complex with the Hsp27 epidermal steroid response element, demonstrating that cucurbitacin is the first well-defined insect steroid hormone antagonist.

20E is an important insect steroid hormone that can bind to a homologous nuclear receptor composed of an ECdysone receptor (EcR) and supercell (USP) to activate the expression of the 20E response gene, leading to subsequent metamorphosis [123]. Zou et al. [124] conducted experiments with Drosophila Kc and silkworm Bm12 cells to screen for 20E antagonists. The experimental results showed that cucurbitacin B could antagonize EcRE activity and gene expression in Kc and Bm12 cells induced by 20E. Cucurbitacin B caused hair loss defects and death in silkworms by antagonizing 20E, as well as skin loss defects and death in Voles melanogaster and cotton bollworms. Additionally, cucurbitacin B inhibited the growth and development of insects by antagonizing 20E. This study provided a reference value for the development of insect growth regulators.

Cowpea is sensitive to nematodes. Biocult and Nemafrici-BL phytofungicides can promote plant growth and inhibit nematode population density, and they can reduce the population density of Bacillus intestinalis in root and soil. Kgabo et al. [125] conducted experiments and found that the combined application of Biocult and Nemafrici-BL have antagonistic effects on the growth of cowpea and should be used separately in the production of cowpea.

Hafiz et al. [109] studied the effects of two concentrations of cucurbitacin B on adult worms (F0) and larvae and their offspring adults (F1), and the results showed that 25 ppm of cucurbitacin B can significantly reduce the adult lifespan and fertility of the F0 and F1 generations, and 100 ppm of cucurbitacin B can reduce the life span and fertility of the F0 generation and the life span of the F1 generation. These results indicate that host plants containing high concentrations of cucurbitacin B may protect themselves from the feeding activities of melon aphids by sublethal damage to their biological adaptability.

## 6. Conclusions and Prospects

After years of development, natural products are still a valuable source of drug discovery. Over the past decade, cucurbitacin B has attracted a great deal of interest as a natural product commonly used in traditional herbal medicines because of its antioxidant, anti-inflammatory, neuroprotective, and anticancer potential. Numerous studies have demonstrated that cucurbitacin B can regulate the cell cycle of various cancer cells through multifunctional pathways, induce autophagy and apoptosis, and exhibit significant anticancer effects in vitro and in vivo, thus attracting considerable attention. A variety of potential targets and signaling pathways related to cucurbitacin B and its derivatives have been identified, and a large number of cucurbitacin B derivatives with different modifications have appeared in front of the world, hoping to find more effective and safer drugs for cancer chemotherapy. It is exciting that in 2022, the research team found a direct action target IGF2BP1 in liver cancer cells, which will provide a feasible reference value for the development and application of cucurbitacin B. However, in order to advance cucurbitacin B and its derivatives into a viable treatment, there are still several questions and new directions for future development to be considered [15].

Firstly, this natural product has received considerable attention in the past, but the exact molecular mechanism of its involvement in the treatment of cancer and other diseases still needs to be further elucidated [126]. In the last decade or so, network pharmacology has been widely used in drug discovery to identify potential molecular mechanisms and therapeutic properties [127]. Some related studies have inspired investigators to identify new and unexplored targets for cucurbitacin B and its derivatives, such as one study demonstrating that cucurbitacin B can inhibit inflammation by inhibiting the IL-6/STAT3/HIF-1α signaling pathway through network pharmacology and in vivo and in vitro experiments [128]. Thus, it has potential protective and therapeutic effects on cholestatic liver injury (CLI), providing a reference value for future drug development and utilization.

Secondly, new drug delivery systems can be developed, which can improve the water solubility of many drugs. This strategy may also improve the water solubility of cucurbitacin B and its derivatives. A preparation of solid liposome nanoparticles of cucurbitacin B created by emulsifying ultrasonic hair increased its activity and decreased its toxicity and dose. Exploring novel drug delivery formulations containing nanosuspensions, nanogels, nanoparticles, micelles, and self-microemulsion systems will provide water-soluble, tumor-targeting, and potent cucurbitinoid Class B drugs for potential clinical applications [129,130,131,132,133].

Thirdly, compared with traditional pesticides, the impact of biopesticides is more moderate. Given the favorable conditions of cucurbitacin B in agriculture, there may be opportunities for it to be developed as a pesticide for agricultural pest control, which is also of great significance for the control and protection of related pests.

Finally, in a large amount of research in the literature, the design and synthesis of cucurbitacin B derivatives have mainly been tested against various cancer cells. In fact, many natural products are multifunctional agents that simultaneously offer opportunities for drug discovery in different therapeutic areas. For example, oleanane triterpene derivative CDDO-Me, which has strong antioxidant, anti-inflammatory, and cytoprotective effects in the low nanomolar range, has significant anti-proliferation and pro-apoptotic effects at relatively high concentrations [134,135]. Based on the multifunctional effects of CDDO-Me, several drugs have been tested in clinical trials, such as chronic kidney disease due to secondary diabetes (stage II), pulmonary hypertension (stage III), and advanced malignant melanoma (stage II). For the above studies, we should also pay attention to exploring other effects of cucurbitacin B, including anti-human immunodeficiency virus activity, antioxidant, blood pressure lowering, cardiac protection, and anti-cryptosporidium generated by cucurbitacin B and its derivatives [136,137,138,139].

As a naturally occurring compound, cucurbitacin B is widely distributed in nature and readily accessible. It serves as a crucial natural product platform for the development of drugs targeting cancer, inflammation, and other diseases. This paper aims to contribute to the research on cucurbitacin B by providing constructive suggestions and opinions for further investigation of its derivatives, thereby accelerating their development and application.

## Figures and Tables

**Table 1:** Cytotoxicity of cucurbitacin B and compounds **1, 2,** and **3** against MCF-7 and Vero cells.

Compounds	IC_50_ (μM)	
	MCF-7	Vero
Cu B	12.0 ± 0.4	0.04 ± 0.02
1	18.1 ± 0.4	12.4 ± 0.5
2	15.4 ± 0.2	1.87 ± 0.24
3	16.6 ± 0.9	0.07 ± 0.02
TAM	22.6 ± 0.3	-

**Table 2:** Antiproliferative activity of cucurbitacin B and compounds **15** and **20**–**27**.

Compounds	IC_50_ (μM)		SI
	HCT116	L02	
**15**	0.031 ± 0.003	0.080 ± 0.010	2.58
**20**	0.051 ± 0.003	0.080 ± 0.008	1.57
**21**	0.034 ± 0.005	0.100 ± 0.005	2.94
**22**	0.063 ± 0.074	0.031 ± 0.005	0.49
**23**	0.076 ± 0.039	0.031 ± 0.004	0.41
**24**	0.121 ± 0.009	0.040 ± 0.000	0.33
**25**	0.044 ± 0.006	0.033 ± 0.011	0.75
**26**	0.037 ± 0.014	0.034 ± 0.004	0.43
**27**	0.079 ± 0.014	0.034 ± 0.004	0.43
**Cu B**	0.019 ± 0.022	0.011 ± 0.003	0.57

**Table 3:** Antiproliferative activity of compound 21 on various cell lines.

Cell Lines	IC_50_ (μM)		SI	
	Cu B	21	Cu B	21
A549	0.088 ± 0.005	0.009 ± 0.002	0.13	11.11
HCT-116	0.019 ± 0.003	0.080 ± 0.010	0.58	1.25
MDA-MB-231	0.037 ± 0.002	0.049 ± 0.005	0.30	2.04
MCF-7	0.040 ± 0.002	0.056 ± 0.003	0.23	1.79
SK-OV-3	0.047 ± 0.004	0.054 ± 0.006	0.23	1.85
HeLa	0.024 ± 0.004	0.015 ± 0.004	0.46	6.67
HepG-2	0.051 ± 0.003	0.080 ± 0.008	0.22	1.25
BXPC-3	0.022 ± 0.001	0.018 ± 0.003	0.50	5.56
PANC-1	0.067 ± 0.008	0.088 ± 0.012	0.16	1.14
CFPAC-1	0.023 ± 0.002	0.037 ± 0.005	0.49	2.70
L02	0.011 ± 0.030	0.011 ± 0.005		

**Table 4:** Activity and TI of cucurbitacin B derivatives **31**–**66** against HepG2 and L02 cell lines.

Compounds	IC_50_ (μM)		TI
	HepG2	L02	
**Cu B**	0.060 ± 0.02	0.019 ± 0.003	0.32
**31**	1.01 ± 0.04	1.02 ± 0.58	1
**32**	0.54 ± 0.04	0.52 ± 0.08	0.96
**33**	2.45 ± 1.75	2.37 ± 0.09	0.97
**34**	4.89 ± 0.92	8.01 ± 0.47	1.64
**35**	9.28 ± 0.37	5.50 ± 2.48	0.59
**36**	0.93 ± 0.06	0.41 ± 0.11	0.44
**37**	0.22 ± 0.11	0.042 ± 0.001	0.19
**38**	9.62 ± 1.34	6.90 ± 1.37	0.72
**39**	7.42 ± 0.40	4.42 ± 0.97	0.60
**40**	4.67 ± 0.28	3.57 ± 2.58	0.76
**41**	2.99 ± 2.06	1.93 ± 0.32	0.65
**42**	3.71 ± 1.69	2.47 ± 0.47	0.67
**43**	0.92 ± 0.44	0.74 ± 0.15	0.8
**44**	4.05 ± 1.39	2.48 ± 0.52	0.61
**45**	3.31 ± 0.24	9.59 ± 2.38	2.90
**46**	2.34 ± 0.91	1.47 ± 0.36	0.63
**47**	3.85 ± 0.63	5.25 ± 1.63	1.36

**Table 5:** Cytotoxic activity of compounds **48**–**65** against HepG2 cells and L02.

Compounds	IC_50_ (μM)		TI
	HepG2	L02	
**Cu B**	0.060 ± 0.02	0.019 ± 0.003	0.32
**48**	0.82 ± 0.33	2.04 ± 0.67	2.49
**49**	0.63 ± 0.29	2.97 ± 0.23	4.71
**50**	0.52 ± 0.09	2.10 ± 1.41	4.03
**51**	0.64 ± 0.26	2.62 ± 0.52	4.1
**52**	1.15 ± 0.24	3.21 ± 0.09	2.79
**53**	0.83 ± 0.38	3.25 ± 0.40	3.92
**54**	1.19 ± 0.55	4.29 ± 0.66	3.61
**55**	1.15 ± 0.24	1.17 ± 0.08	1.02
**56**	6.92 ± 0.75	3.27 ± 0.43	0.47
**57**	10.28 ± 1.11	6.69 ± 3.95	0.65
**58**	-	-	-
**59**	1.01 ± 0.40	2.72 ± 0.38	2.69
**60**	1.35 ± 0.10	2.94 ± 0.09	2.18
**61**	1.11 ± 0.22	3.31 ± 0.18	0.34
**62**	1.02 ± 0.28	3.00 ± 0.26	2.94
**63**	0.46 ± 0.14	0.69 ± 0.43	1.5
**64**	0.53 ± 0.01	0.80 ± 0.40	1.51
**65**	0.92 ± 0.16	2.61 ± 1.77	2.84

**Table 6:** Cytotoxic activity of compounds **67**–**95** against A549 cells.

Compounds	IC_50_ (nM)
	A549
**Cu B**	12.3 ± 2.3
**67**	61.0 ± 6.8
**68**	171.6 ± 37.3
**69**	34.5 ± 2.1
**70**	13.0 ± 1.3
**71**	44.4 ± 3.6
**72**	97.2 ± 21.6
**73**	30.6 ± 7.8
**74**	61.3 ± 11.4
**75**	23.3 ± 4.8
**76**	36.7 ± 9.8
**77**	13.2 ± 2.6
**78**	46.0 ± 8.4
**79**	35.3 ± 6.1
**80**	11.6 ± 1.9
**81**	10.7 ± 1.9
**82**	17.7 ± 2.1
**83**	8.5 ± 1.0
**84**	88.1 ± 19.5
**85**	81.4 ± 13.4
**86**	16.2 ± 2.7
**87**	6.8 ± 0.9
**88**	47.7 ± 10.3
**89**	160.1 ± 31.2
**90**	1460 ± 310
**91**	25.7 ± 6.7
**92**	14.4 ± 4.6
**93**	29.2 ± 6.4
**94**	49.7 ± 8.4
**95**	16.5 ± 4.6

**Table 7:** Inhibitory effect of cucurbitacin B analogues on the proliferation of A549 cells.

Compounds	IC_50_ (μM)		Increase Toxicity (CC_48_/CC_72_)
	48 h	72 h	
**Cu B**	19.91	12.09	1.6
**100**	54.42	26.49	2.1
**101**	-	-	-
**102**	-	-	-
**103**	-	-	-
**104**	14.65	2.64	5.5
**105**	-	-	-
**106**	28.80	11.52	2.5
**107**	1.33	0.12	1.2
**108**	0.42	0.12	11.1
**109**	-	-	-
**110**	14.65	6.90	2.1
**111**	-	-	-
**112**	-	-	-
**113**	-	-	-
**114**	-	-	-
**115**	-	-	-
**116**	-	-	-
**Doxorubicin**	3.69	1.18	3.1
**Paclitaxel**	1.16	0.19	6.1

## Data Availability

Data sharing is not applicable to this article as no new data were created or analyzed in this study.
